# High Dietary Folic Acid Intake Is Associated with Genomic Instability in Peripheral Lymphocytes of Healthy Adults

**DOI:** 10.3390/nu14193944

**Published:** 2022-09-23

**Authors:** Khadijah I. Alnabbat, Ali M. Fardous, Aiman Shahab, Andrew A. James, Manhel R. Bahry, Ahmad R. Heydari

**Affiliations:** 1 Department of Nutrition and Food Science, Wayne State University, Detroit, MI 48202, USA; 2 Department of Food and Nutrition Sciences, King Faisal University, Al Hufūf 31982, Saudi Arabia; 3 Barbara Ann Karmanos Cancer Institute, Wayne State University, Detroit, MI 48202, USA

**Keywords:** folate, folic acid, excess, UMFA, lymphocyte, micronuclei, fortification, methylation

## Abstract

Mandatory fortification of food with synthetic folic acid (FA) was instituted in 1998 to reduce the incidence of neural tube defects. Adequate folate status is correlated with numerous health benefits. However, elevated consumption of FA is controversially associated with deleterious effects on health. We previously reported that excess FA mimicked folate depletion in a lymphoblastoid cell line. To explore the impact of FA intake from fortified food, we conducted an observational human study on 33 healthy participants aged 18–40 not taking any supplements. Food intake, anthropomorphic measurements, and blood samples were collected and analyzed. Our results show that individuals belonging to the highest tertile of folic acid intake, as well as ones with the highest folic acid to total folate intake ratio (FAR), display a significantly greater incidence of lymphocyte genomic damage. A decrease in global DNA methylation is observed in the highest tertile of FAR compared to the lowest (*p* = 0.055). A downward trend in the overall gene expression of select DNA repair and one carbon cycle genes (MGMT, MLH1, UNG, MTHFR, MTR) is noted with increased folate status and FA intake. These results provide supporting evidence that high consumption of FA from fortified foods can precipitate genomic instability in peripheral lymphocyte in vivo.

## 1. Introduction

Folate is an essential vitamin present naturally in green vegetables, liver, legumes, and some fruits. It acts as cofactor for several enzymes involved in DNA biosynthesis, repair, and maintenance. Impaired folate metabolism and folate deficiency have been linked to neural tube defects (NTD) [1], carcinogenesis in a wide range of tissues [2,3,4], and are implicated in the pathogenesis of diseases such as cardiovascular disease and neurocognitive disorders [5]. Several countries including USA, Canada and Chile have mandated fortification programs of grains and grain products with folic acid (FA), a synthetic version of folate, to mitigate NTD [5,6]. These efforts led to an increase in population folate intake and a marked reduction in NTDs [7,8]. An unintended consequence of this fortification effort is the current prevalence of total folate intake exceeding the upper limit (UL) of 1 mg/day [9], with many surpassing the recommended daily allowance (RDA) due to a combination of consumption of fortified food and voluntary supplementation [10,11,12,13,14]. Central to this observation is the emerging evidence that folic acid, a stable, highly bioavailable, synthetic variant of folate, could modulate health and disease differently than natural folates [5,15,16,17]. Elevated FA levels are known for masking and exacerbating vitamin B12 deficiency [5,9,18], and are controversially implicated in increased cancer onset, progression, and mortality rate [5,18,19,20]. The controversies surrounding FA supplementation extend to a number of other diseases and conditions such as metabolic disorders [21], immunity [22,23], colon cancer [20,24,25], and autism spectrum disorders [26,27].

**Figure 1:**
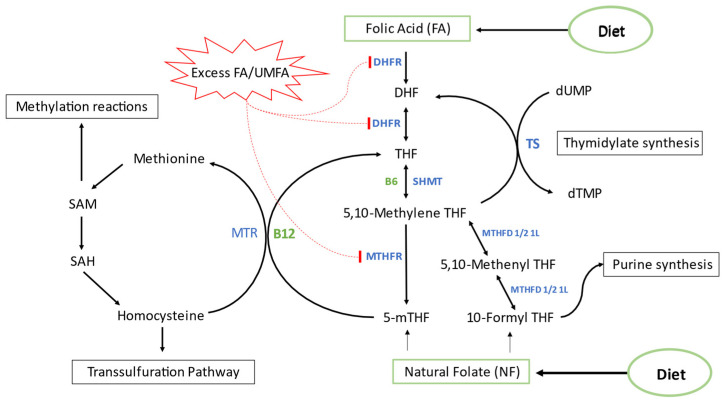
One carbon folate metabolism. Abbreviations: FA = Folic acid, NF = Natural Folate, UMFA = unmetabolized folic acid, DHFR = Dihydrofolate reductase, DHF = Dihydrofolate, THF = Tetrahydrofolate, SHMT = Serine hydroxymethyltransferase, TS = Thymidylate synthase, MTHFR = Methylenetetrahydrofolate reductase, MTHFD = Methylenetetrahydrofolate dehydrogenase, MTR = 5-Methyltetrahydrofolate-Homocysteine Methyltransferase or Methionine synthase, SAM = S-adenosyl-methionine, SAH = S-adenosyl-homocysteine.

Folate exists naturally as 5-methyl-tetrahydrofolate (5-mTHF) or 5-formyl-tetrahydrofolate (5-fTHF), which also can be converted rapidly and efficiently by human intestinal mucosa to 5-mTHF. Unlike folate, FA is required to be activated and converted to 5-mTHF mainly by dihydrofolate reductase (DHFR) and methylene tetrahydrofolate reductase (MTHFR) in a multi-step reaction [28,29,30]. This process of activation is slow and rate limiting in humans causing FA to appear in the blood as unmetabolized FA (UMFA) and persisting in circulation for 12 h after fasting indicating poor handling by human tissues [31,32]. Reports from the United States reveal that UMFA is present in the majority of analyzed population samples [11,33,34,35]. Daily intake of FA exceeding 200 µg is positively correlated with chronically elevated UMFA levels in a dose dependent manner [11,18,33]. UMFA is capable of acting as a competitive or non-competitive inhibitor of DHFR depending on intracellular dihydrofolate (DHF) concentration [31]. DHF is generated naturally during thymidylate synthesis, and it was found that DHFR shares transcription factors with thymidylate synthase (TS) [36,37]. Chronic inhibition of DHFR by UMFA could lead to the accumulation of DHF, a potent inhibitor of MTHFR, potentially leading to a disruption of folate metabolism and the one carbon cycle [38,39,40] (Figure 1).

We recently reported that excess folic acid can mimic folate deficiency in a human lymphoblastoid cell line (LCL) by similarly impacting genomic damage, global methylation, and expression of DNA repair genes [41]. Others reported complementary findings in *C. elegans* and mouse models where both FA deficiency and excess supplementation led to deleterious effects [42,43,44]. Lymphocytes are particularly useful for studying the effects of micronutrient intake on genomic stability. Lymphocyte total folate is highly sensitive to dietary folate intake and correlates with plasma 5-mTHF and homocysteine, but not with RBC folate, indicating that lymphocytes could be a better indicator of acute changes in dietary folate and micronutrient intake [45]. The impact of folate status on lymphocytes can be assessed through validated and sensitive markers such as micronuclei (MNi), and DNA methylation [30,45,46,47,48]. Therefore, we designed this human observational study to characterize the in vivo impact of dietary FA from food fortification on lymphocyte genetic stability using specific markers such as MNi through the cytokinesis block micronuclei assay (CBMN), and global methylation using the Line 1 Methylation assay.

## 2. Materials and Methods

### 2.1. Recruitment and Data Collection

**Figure 2:**
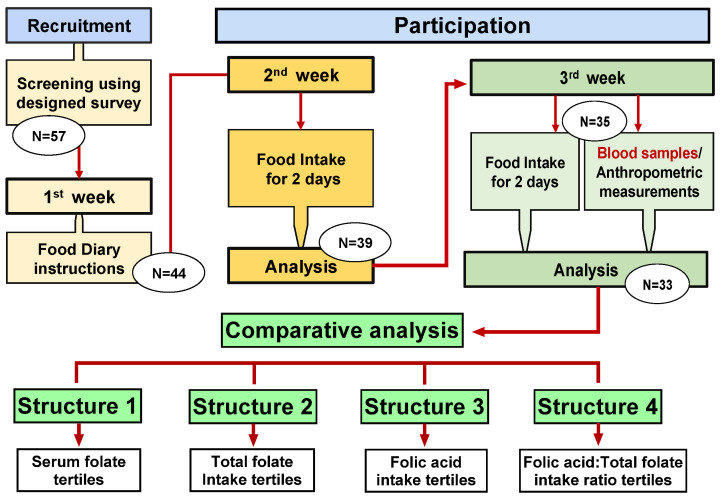
Study design schematic. Fifty-seven healthy adults aged (18–40) were recruited for the study. Participants were initially screened, followed by food diary collection, analysis, and screening, additional food intake analysis, blood sampling, and collection of data. Data analysis was performed using 4 comparison structures based on: serum folates tertiles (structure 1), total folate intake tertiles (structure 2), folic acid intake tertiles (structure 3), and folic acid/total folate intake ratio tertiles (structure 4).

A total of 57 individuals were recruited for this study using flyers that were distributed across the campus at Wayne State University. A food folate survey was designed and validated by comparing survey data to 24 h dietary recall across 10 individuals. Survey was subsequently used to screen participants and estimate individual’s food and folic acid intake (Supplemental Table S1). Our inclusion criteria were healthy adult between the ages 18–40. Exclusion criteria included those undergoing medical treatment, taking medications or drugs, pregnant and lactating women, strict vegetarian or vegan, heavy alcohol intake, smokers, and B-vitamins, folic acid, or multivitamin supplementation. At the first encounter, participants read and signed informed consent forms. food intake diary instructions were given by trained personnel and participants were asked to record food intake for two days (one weekday and one weekend) in the first week. Participants that met the study’s guidelines were asked to provide an additional 2 days of food intake the following week and donate blood samples. Participants anthropometric measurements were obtained and recorded. Thirty-three individuals met all the inclusion criteria for the study and were included in the final analysis (Figure 2). This study was approved by the Institutional Review Board (IRB), Wayne State University, Detroit, MI.

### 2.2. Dietary Intake Assessment and Folic Acid Intake Analysis

Dietary intake was assessed using a 4-day food diary over two weeks (2 weekdays and 2 weekend days). Participants were asked to indicate details about the food item, name, type, size, amount, labeling, recipe for home-cooked item, or the name of restaurant. Food diaries were reviewed with participants by trained dietitians to ensure accuracy. Food diaries were carefully audited, and details were verified by contacting participants when necessary, examining food labels, and contacting restaurants. Food intakes were then analyzed using ESHA food processor nutrition analysis software V11.10 (ESHA Research, Salem, OR, USA) [49] to obtain macronutrient (protein), micronutrient (Iron, choline, B1, B2, B3, B6, B12, Folate) intake. However, since the software does not discriminate between natural folate and folic acid, we obtained the amount of folic acid from fortified food by matching the food items to the USDA Food Data Central (https://fdc.nal.usda.gov/, accessed on 1 October 2020) [50]. Matching of the food items from Food Data Central was carefully performed to reflect the same type, ingredients, and portion size as the items analyzed in ESHA to ensure consistency.

### 2.3. Blood Samples Collection and Analysis

Blood collection was performed the morning of the study following an overnight fast and before having breakfast. Participants were encouraged to stay hydrated to avoid misleading complete blood count results. Blood samples were collected and individually aliquoted (seven × 4 mLs k_2_ EDTA tubes, one × 10 mL silicone coated red tube, and two × 5 mLs silica/polymer gel (serum separator) tubes) by a certified phlebotomist. Samples were then quickly processed for analysis in our laboratory (Cytome biomarkers, LINE methylation assay, gene expression) or in a certified medical laboratory [51]; (serum folate (SF), RBC folate, plasmatic homocysteine (Hcy), Serum B12, methylmalonic acid (MMA), plasmatic B2, B6, and Complete blood count (CBC)).

### 2.4. Cytokinesis-Block Micronucleus (CBMN) Assay

The cytokinesis block micronucleus assay (CBMN) measures endpoints of DNA damage, such as micronuclei (MNi), nucleoplasmic bridges (NPBs), and nuclear buds (NBUDs); collectively known as cytome biomarkers. The CBMN assay and its associated markers are robust and sensitive indicators of small changes in micronutrient status. This method has been used extensively to elucidate the impact of folate status on genomic stability in lymphocyte [46,47,52,53]. Lymphocytes were isolated within 2 h of collection using a density gradient medium (Lymphoprep TM, Stem Cell technologies, Cologne, Germany). The lymphocytes were subjected to the CMBN assay protocol adopted form the work of Thomas and Fenech [48,54]. Briefly, the isolated lymphocytes were washed twice in Hank balanced salt solution (HBSS), and then resuspended in culture media. Cell concentration was estimated using automated cell counter. Cells were cultured at concentration of 1 × 10^6^ cells/mL in RPMI 1640 medium with 10% FBS, 1% penicillin, 1% glutamax and 300 nM FA. All cultures were prepared in duplicate. Forty-four hours after phytohemagglutinin (PHA) stimulation (45 µg/mL), cytochalasin-B (4.5 mg/mL) was added, and cells were harvested on slides 28 h later. Cells were harvested in duplicate using Shandon Cytospin 4 (Thermo Scientific, Waltham, MA, USA) at 600 rpm for 5 min. slides were air-dried, fixed in absolute methanol, and stained using Shandon Kwik-Diff Stains (Thermo Scientific, Waltham, MA, USA). Slides were cover-slipped using DPX mountant (Sigma Aldrich, St. Louis, MO, USA). The frequency of MNi, NPB and NBUD was determined in 2000 binucleated (BN) cells following the scoring criteria of the HUMN project guidelines [55] by two trained scorers in a blinded manner as previously described [41].

### 2.5. LINE-1 Methylation Assay

Genomic DNA was isolated within 2 h of collection using PureLink^®^ Genomic DNA Mini Kit (life technologies, Carlsbad, CA, USA) following the manufacture protocols. LINE-1 methylation assay was performed using Global DNA Methylation LINE-1 Kit (Active Motif, Carlsbad, CA, USA). One hundred ng Msel digested genomic DNA was hybridized to the LINE-1 probe, immobilized to a streptavidin-coated plate, incubated with primary and secondary antibodies, then analyzed through a colorimetric plate reader reaction. Data were obtained by comparing to a standard curve of methylated and non-methylated DNA.

### 2.6. Gene Expression Profiling

Blood was collected in K_2_-EDTA tube, and RNAlater (Ambion, Austin, TX, USA) was added to the samples to prevent mRNA degradation. Total RNA was extracted from blood within 4 h of collection using RiboPure^TM^-Blood kit (Ambion, Austin, TX, USA). DNase I digestion (8 U/µL) was performed to remove contaminating genomic DNA. cDNA was synthesized using the ImProm-II^TM^ Reverse Transcription System (Promega, Madison, WI, USA). Gene expression levels were quantified with quantitative real time PCR (qPCR), PikoReal 96 (Thermofisher, Vantaa, Finland) and normalized to the geometric mean of HPRT1 and ß-Actin using the 2^−(∆∆Cq)^ method. Primers were validated and tested using external standards for each gene prepared by subcloning using the TOPO^®^ TA Cloning^®^ kit (Invitrogen, Carlsbad, CA, USA). Primer sequences are provided in Supplemental Table S6.

### 2.7. Statistical Analysis

Tabulated data are presented as mean (standard deviation). Mean comparison between two groups was performed using student *t*-test. One-way ANOVA was used to compare mean of 3 groups with Tukey post hoc test analysis where appropriate. Multivariate and principal component analysis (PCA) was performed on log transformed data (data were not normally distributed). Pearson’s correlation coefficient was used to evaluate the correlation level between two variables. *p*-value < 0.05 was considered statistically significant. Data were analyzed using SPSS 25.0 (IBM, Armonk, NY, USA).

## 3. Results

### 3.1. General Participants Characteristics

The demographic and anthropometric measurements characteristics of the subjects in the study are listed in (Supplemental Table S2). The analysis was performed on 33 healthy individual comprising of 21 males and 12 females with a mean age 30.8 years (fifty-seven participants were initially recruited, 14 individuals met the exclusion criteria or dropped out). Females mean body mass index (BMI) was in the normal range, while mean male BMI was in the overweight range. However, both male and female waist to hip ratio (WHR) values fell in the low-health risk ratio of developing cardiovascular diseases.

### 3.2. Systemic Markers

Mean serum folate, B12, plasma B2 and B6 fell in the reference range. B12, MMA, and Hcy levels are sensitive indicators of a functional B12 deficiency. While mean values for B12 and MMA fell within normal ranges, mean plasma homocysteine was elevated (Supplemental Table S3). Within our study population, 39% had moderate Hcy level (15–20 µmol/L), 33% high Hcy level (>20 µmol/L), and 18% had high serum folate (>20 µg/L).

### 3.3. Nutrient Intake

We evaluated the individual intake of protein, choline, iron, B1, B2, B3, B6, B12, and folate using recommended dietary intake values adjusted for gender, and age. The mean intake is shown in (Supplemental Table S4) with RDA and AI reference. Our food diary analysis showed that the participants on average met or exceeded the RDI for micronutrient intake except for choline.

### 3.4. Comparative Data Analysis

To characterize the effects of dietary natural folate and folic acid intake on peripheral lymphocytes genomic stability, we arranged the data in four structures. The structures were generated based on the tertiles of: serum folate µg/L (structure 1; T1 < 13, 13 < T2 < 18, T3 > 18), total folate intake expressed as µg dietary folate equivalent (DFE) (structure 2; T1 < 400, 400 < T2 < 600, T3 > 600), folic acid intake expressed in µg DFE (structure 3; T1 < 100, 100 < T2 < 200, T3 > 200) and folic acid to total folate ratio (FAR) (structure 4; T1 < 0.3, 0.3 < T2 < 0.5, T3 > 0.5). data tables are provided in Supplementary Table S5.

### 3.5. Cytome Biomarkers Analyses

**Figure 3:**
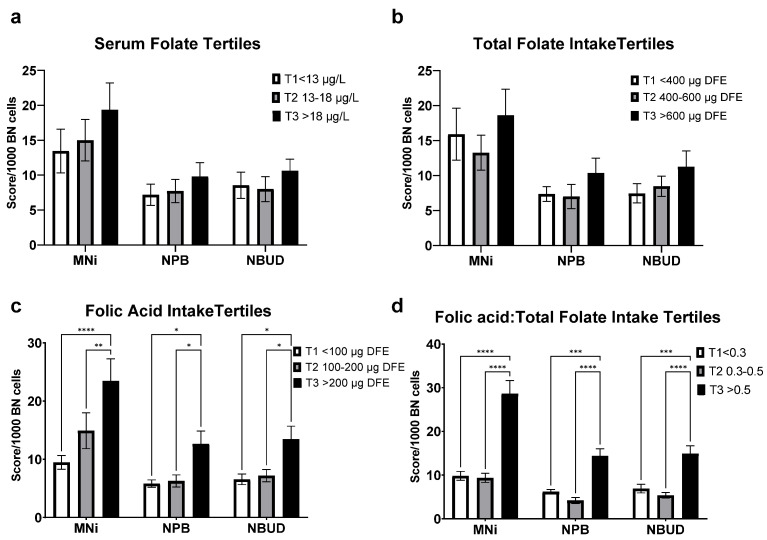
Frequency of Cytome biomarkers in human lymphocytes relative to: (**a**) serum folate tertiles 1. (**b**) Total dietary folate intake tertiles. (**c**) Dietary folic acid intake tertiles. (**d**) Folic acid intake: total folate intake ratio tertiles. Serum folate presented as µg/L (ng/mL), DFE; (dietary folate equivalent), data presented as mean (±SEM), *n* = 33, * *p* < 0.05, ** *p* < 0.005, *** *p* < 0.001, **** *p* < 0.0001.

There was no significant association between cytome biomarkers and folate status in the analysis of structure 1, based on serum folate, and structure 2, based on total folate intake (Figure 3a,b). structure 3, based on folic acid intake revealed that mean MNi score of the highest tertile (>200 µg DFE) is significantly different than the 2nd and 1st tertile (100–200 and <100 µg DFE, *p* < 0.005 and *p* < 0.0001, respectively) (Figure 3c). Structure 4, based on folic acid to total folate intake ratio (FAR), revealed a clear separation of all cytome biomarkers between the third, and the first and second tertiles (*p* < 0.001) (Figure 3d).

### 3.6. LINE-1 Methylation

**Figure 4:**
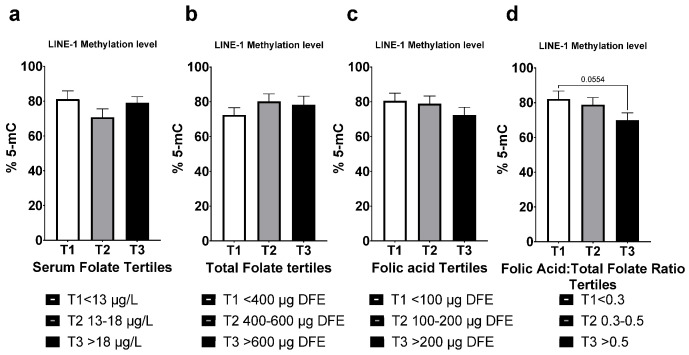
Global LINE-1 methylation levels in human lymphocytes based on: (**a**) serum folate tertiles 1. (**b**) Total dietary folate intake tertiles. (**c**) Dietary folic acid intake tertiles. (**d**) dietary folic acid intake: total folate intake ratio tertiles. Serum folate presented as µg/L (ng/mL), DFE; (dietary folate equivalent), data presented as mean (±SEM), *n* = 33.

The LINE-1 methylation Assay detects methylated sites in the long interspersed nuclear elements. These mobile parasitic elements comprise 17% of the human genome and its methylation status is used as a surrogate marker for global genomic methylation. We observed a downward trend in mean lymphocyte methylation level in the third tertile (*p* = 0.055) compared to the first in structure 4, based on FAR (Figure 4d). No significant differences were observed in the other comparison structures.

### 3.7. Gene Expression

**Figure 5:**
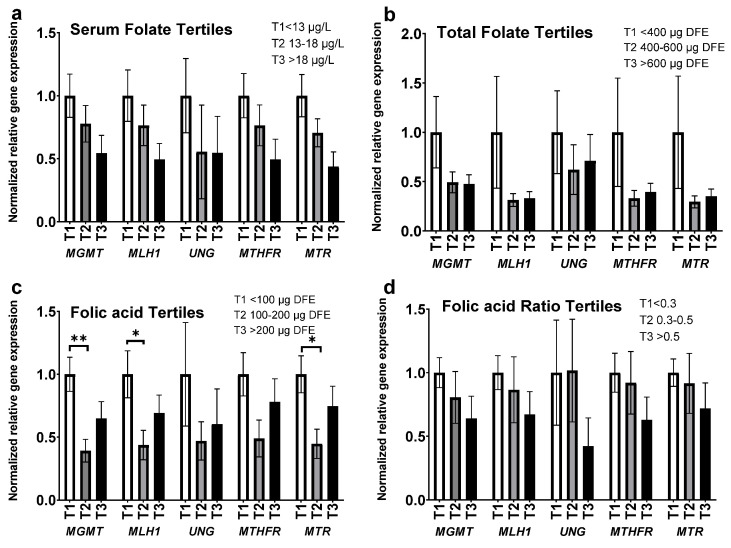
Normalized relative gene expression of O-6-methylguanine-DNA methyltransferase (*MGMT*), mutL homolog 1 (*MLH1*), uracil DNA glycosylase (*UNG*), 5,10-methelenetetrahydrofolate-reducatase (*MTHFR*), and 5-methyltetrahydrofolate-homocysteine methyltransferase (*MTR*) relative to: (**a**) serum folate tertiles 1. (**b**) Total dietary folate intake tertiles. (**c**) Dietary folic acid intake tertiles. (**d**) Dietary folic acid intake: total folate intake ratio tertiles (FAR). Serum folate presented as µg/L (ng/mL), DFE; (dietary folate equivalent), data presented as mean (±SEM), *n* = 33, * *p* < 0.05, ** *p* < 0.005.

Folate status impacts various critical genes involved in DNA damage and repair. We performed gene expression analyses on the following genes; O-6-methylguanine-DNA methyltransferase (*MGMT*), mutL homolog 1 (*MLH1*), uracil DNA glycosylase (*UNG*), 5,10-methelenetetrahydrofolate-reducatase (*MTHFR*) and 5-methyltetrahydrofolate-homocysteine methyltransferase (*MTR*), which is also known as methionine synthase (*MS*). *MGMT*, *UNG*, *MLH1* are genes encoding proteins that participates in DNA damage repair, while both *MTHFR* and *MTR* participate in folate metabolism and folate-methylation flux. We observed a downward trend in gene expression between the highest and lowest tertiles based on serum folate levels, total folate intake, and FAR (Figure 5a,b,d). A similar trend was observed between the tertiles based on folic acid intake levels with a significant decrease noted between the 1st and 2nd tertiles with *MGMT*, *MLH1*, and *MTR* (*p* < 0.005, *p* < 0.05, *p* < 0.05, respectively) (Figure 5c). Two-way ANOVA analysis was performed to determine the effects of folate status, and gene selection, on variations between tertiles. A simple main effect analysis showed that serum folate levels, folic acid intake levels, and FAR had a significant impact on the observed differences in gene expression between tertiles (*p* < 0.0003, *p* < 0.0003, *p* < 0.03, respectively), while total folate levels had no significant effect.

## 4. Discussion

Folate’s role in nucleotide synthesis, DNA repair, and genomic stability is well established. Several studies evaluated the impact of folate deficiency and adequacy on MNi, NPB and NBUD formation in human lymphocytes in vitro [41,56,57,58,59]. Based on the current body of evidence, the prevailing paradigm suggests that folate deficiency is deleterious, while a high folate intake is beneficial. However, in vivo studies evaluating either serum folate, RBC folate, homocysteine level or folate intake, and the resulting association with cytome biomarkers found conflicting results [46,47,55,60]. It is important to note that many published reports did not differentiate between natural and synthetic folate intake. In fact, many in the field inadvertently use the term folate to describe both natural and synthetic forms, potentially leading to misinterpretation of results. We previously demonstrated that excess folic acid could mimic folate deficiency in a human lymphoblastoid cell line (LCL) [41]. To date, the effects of excessive folic acid intake on cytome biomarkers in human lymphocytes in vivo has not been investigated. Therefore, the primary aim of this study was to determine whether high intake of folic acid through the consumption of fortified food is associated with lymphocyte genome instability in healthy adults.

There is emerging evidence that the synthetic folic acid may impact health and disease differently than natural folate [15,16]. FA is highly stable and bioavailable compared to natural folates, leading to enhanced absorption and elevated systemic levels compared to natural folates [61]. Following the food fortification mandate, studies reported that total folate intake exceeding the upper limit (UL) of 1 mg/day, is now prevalent in the United States [9]. Results from the NHANES 1999–2000 study revealed that 23% of the population and 43% of all children had elevated serum folates [62]. Unsurprisingly, UMFA was detected in most samples collected from US children, adolescent, and adults [11,33,34,35]. Controversially, UMFA can impair folate metabolism by inhibiting DHFR and MTHFR, potentially leading to a functional folate deficiency [39,40]. Others suggested that the biological effects of UMFA are mediated through non-canonical pathways independently of folate mediated one carbon metabolism [63]. Findings from multiple studies confirm that UMFA concentrations are closely associated with folic acid intake, 5-mTHF, and total folate concentrations [33,34,64]. A summary report form a recent NIH workshop highlighted significant knowledge gaps in our understanding of the effects of excess folic acid and UMFA on human health [63]; Despite a growing body of evidence suggesting UMFA may play a role in the potential adverse effects of excess FA on health, there is a current lack of strong mechanistic and biological link with such effects. While our study did not quantify UMFA levels and examine the association with lymphocyte genetic instability, future mechanistic studies should carefully investigate the role of UMFA in excess FA associated pathologies.

In our previous work on LCLs, we observed that high levels of FA could mimic the effects of folate deficiency, precipitating DNA damage, and impacting DNA methylation, and DNA repair in vitro [41]. To assess the impact of FA in vivo, we evaluated the association between serum folate, total folate intake, folic acid intake, and folic acid ratio to total folate intake (FAR), on DNA damage repair through the frequency of cytome biomarkers in peripheral lymphocytes. Our analyses indicated that neither serum folate nor RBC folate correlated with cytome biomarkers. Likewise, we did not detect a significant correlation between serum folate, total folate intake, and cytome biomarkers. Our results agree with and corroborate the finding by Fenech and colleagues [46,47,59]. However, other studies reveal contradicting results that support associations between folate intake and DNA damage in lymphocytes [53,60]. It is difficult to draw parallels and compare the results of these studies due to varying designs, study approaches, and confounding factors such as pre-existing deficient folate status, concomitant micronutrient deficiencies, co-morbidities, age, and other factors. Serum folate levels are known to correlate strongly with total folate intake, and both parameters do not distinguish between natural folate or the synthetic form FA [45,65]. A study by Ladeira et al. that used food frequency questionnaire found no significant correlation between total folate intake and MNi, NPB and NBUD [66]. Our results reveal a significantly higher MNi, NPB and NBUD frequencies in the highest tertile of FA intake (>200 mg DFE). High MNi, NPB and NBUD frequencies were also observed in the highest tertile of FAR (>0.5) when compared with second and first tertiles (0.35 > T2 < 0.5, T1 < 0.35). interestingly a lower ratio of folic acid to natural folate (FAR < 0.5) appeared to be protective signifying that a high intake of natural folate proportional to folic acid is beneficial. The significance of our findings stems from the scarcity of published data isolating the effect of FA intake alone as food fortificant on genomic damage in humans. These findings provide additional support to the notion that excessive FA intake could induce a phenotype similar to that of folate deficiency. In fact, published work using the *C. elegans* model revealed that high levels of FA induced oxidative stress and increased Hcy levels [42], while others showed that both FA deficiency and excess similarly impaired folate metabolism [43]. Similarly, Both FA deficiency and Supplementation impaired hematopoiesis and folate-dependent biosynthetic pathways in mouse B-lymphocytes [44]. It would be of great interest for future studies to test whether the FA induced insufficiency can be resolved by supplementing folate in the natural forms such as 5-mTHF or 5-fTHF. Unlike FA, 5-mTHF and 5-fTHF do not require DHFR to be activated [67], and therefore are unaffected by the inhibition of DHFR by excess folic acid. Natural folate can contribute to the synthesis of nucleotides even in the absence of DHFR activity, and subsequently normal DNA replication, repair, and RNA transcription processes can proceed [68]. 5-mTHF can donate a methyl group once entering the cell in a B12 dependent reaction to convert homocysteine to methionine independently of DHFR activity [29].

It is inviting to suggest that the underlying mechanism of MNi formation observed can be attributed to hypomethylation of DNA. Although we did not detect a statistically significant difference in LINE-1 methylation between the 1st and third FAR tertiles (*p* = 0.055), a trend was observed whereas the third tertile has the lowest methylation level. LINE-1 hypomethylation is associated with increasing MNi frequency in human lymphocytes [69]. CpG hypomethylation was shown to be associated with MNi formation in healthy young males [47]. Charles et al. showed that supraphysiological level of FA induces LINE-1 hypomethylation in a tissue specific, and passage dependent manner [70]. Work in *C. elegans* by Ortbauer et al. suggests that both folate deficiency and FA over-supplementation disrupt the folate cycle by favoring thymidylate synthase over methionine synthase, implying that at either extreme of FA status, nucleotide synthesis is favored over methylation reactions [43]. Hypomethylation can occur when UMFA can potentially saturate, and in turn inhibit the biotransformation of FA to 5-mTHF by DHFR and MTHFR, which is essential for the regeneration of the universal methyl donor S-Adenosyl-methionine (SAM) [39,40]. In a mouse model, FA supplementation resulted in an inhibition of MTHFR gene and protein expression, effectively reducing 5-mTHF concentration [71]. Similarly, a recent report revealed that FA supplementation in mice resulted in altered choline and methyl metabolism, and downregulation of MTHFR in both mother and progeny [72]. Recent Human studies did not find an association between folate status, folic acid intake, and DNA methylation [73,74]. However, others revealed a link between high folate intake in mothers and insulin resistance and adiposity in children, suggesting an epigenetic link [21,75]. In fact, in rodent studies, high maternal FA intake is linked to metabolic and behavioral changes in the offspring suggesting a significant role of FA in precipitating inheritable epigenetic changes through alteration of methylation and imprinting [72,76,77,78,79]. The link between excess FA and the effects on gene specific and global methylation remains an open question that requires additional investigation to fully elucidate the impact on human health.

Elevated homocysteine levels are an established risk marker for cardiovascular disease, stroke, neurodegenerative disease, and systemic inflammation. Hcy levels correlate positively with MNi scores in lymphocytes of adult males [46,47]. We did not detect a significant association in our study between Hcy levels and cytome biomarkers. Mean Hcy levels were consistently lower in the third tertile compared to the first tertile in all comparison structures but only reached statistical significance in the first structure based on serum folate (Supplemental Table S5). Our results suggest that the effect of FA intake on lymphocyte genomic instability may be independent of Hcy. However, further research is needed as little is known about the effects of chronic exposure from excess dietary FA on Hcy levels. Preliminary evidence from *C. elegans* studies suggest that UMFA resulting from excessive FA can lead to saturation of DHFR and increased Hcy levels [42]. Interestingly, Hcy levels was found to stabilize as the RBC folate levels increased, only to rise again when RBC folate level exceeded the reference range in an elderly population with Alzheimer disease [80].

A study from Chile where folic acid fortification of grains is mandatory revealed that high level of circulating folate was associated with DNA methylation of the promoter regions of *MGMT*, and *MLH1* [81]. Aberrant methylation of promoter region of tumor suppressor genes is linked to decreased gene expression or inactivation and is associated with defects in DNA repair and cancer [82]. Folate deficiency is associated with increased uracil misincorporation into the DNA leading to DNA damage and chromosomal breakage [83]. *UNG* is a glycosylase that removes uracil form the DNA constituting the first step in the base excision repair pathway, while *MGMT* and *MLH1* play an important role in preventing and correcting DNA mismatch errors during replication and transcription. We observed a downward trend in the relative gene expression of *MGMT*, *MLH1*, and *UNG* with increased levels of serum folate, folic acid intake, and FAR. The same downward trend was also observed with the critical one carbon cycle enzymes *MTHFR* and *MTR.* This is consistent with published reports where high FA intake was associated with reduced *MTHFR* gene expression, protein, and activity levels in mice [71,84]. Reduction of MTHFR and *MTR* activity is associated with decreased methylation potential and perturbation of DNA methylation reactions. While our results do not elucidate a clear link between the observed gene expression trends and the impact of FA on genomic stability and other possible deleterious effects, it does however highlight the potential of excess FA to alter critical genes and pathways.

## 5. Conclusions

Our data reveal a link between increased folic acid intake and genomic instability in lymphocytes of healthy adults. We show that a higher folic acid intake relative to natural folate leads to increased Cytome biomarkers and possibly reduced DNA methylation in peripheral lymphocytes in vivo. Despite the inherent limitation in our study design and size, the correlations observed compel us to investigate these effects further in larger observational and interventional studies. It would be of utmost importance for public health to elucidate whether the increased exposure to synthetic FA in the human population through fortification and supplementation can lead to deleterious effects.

## Data Availability

The datasets generated during and/or analyzed during the current study are available from the corresponding author on reasonable request.

## Supplementary Materials

The following supporting information can be downloaded at: https://www.mdpi.com/article/10.3390/nu14193944/s1, Table S1: Food Survey, Table S2: General participants characteristics, Table S3: Mean systemic markers, Table S4: Nutrient intake, Table S5: Proposed comparison structures, Table S6: Qiagen-RT2 qPCR Primers.

## Abbreviations

5-fTHF	5-formyl-tetrahydrofolate
5-mTHF	5-methyl-tetrahydrofolate
CBMN	Cytokinesis block micronuclei assay
DHF	Dihydrofolate
DHFR	Dihydrofolate reductase
FA	Folic acid
FAR	Folic acid to total folate intake ratio
Hcy	Homocysteine
LCL	Lymphoblastoid cell line
LINE-1	Long interspersed nuclear elements
MGMT	O-6-methylguanine-DNA methyltransferase
MLH1	mutL homolog 1
MNi	Micronuclei
MMA	Methylmalonic acid
MTHFR	Methylene tetrahydrofolate reductase
MTR	5-methyltetrahydrofolate-homocysteine methyltransferase
NBUD	Nuclear buds
NPB	Nucleoplasmic bridges
NTD	Neural tube defects
RBC	Red blood cells
RDA	Recommended daily allowance
TS	Thymidylate synthase
UL	Upper limit
UMFA	Unmetabolized folic acid
UNG	Uracil DNA glycosylase
