# Supplementary material for: High Dietary Folic Acid Intake Is Associated with Genomic Instability in Peripheral Lymphocytes of Healthy Adults
Source: Nutrients. 2022 Sep 23;14(19):3944. doi: 10.3390/nu14193944 (PMC9571807; doi:10.3390/nu14193944)
Supplement: Supplementary file 1 [file nutrients-14-03944-s001.zip › nutrients-1903517-supplementary.pdf]

Supplementary Materials

High dietary folic acid intake is associated with genomic instability in peripheral lymphocytes of healthy adults.

Khadijah I. Alnabbat[1,2,†], Ali M. Fardous[1†], Aiman Ismail[1], Diane C. Cabelof[1], and Ahmad R. Heydari[1,3,*]

Supplemental Table S1: Food Survey

Q1:	How do you describe your diet style?<br><br>Vegan Vegetarian Protein-rich Typical
Q2:	How do you describe your daily food intake?<br><br>Mostly Carbs. Mostly proteins Mostly fruit and vegetables Balanced
Q3:	How often do you consume these foods per week?

Foods	None	1X	2-3X	≥ 4 X	Can you tell the amount?
Ready to eat breakfast					
Pancakes, waffles					
Breakfast cereals					
Oatmeal					
Cakes, muffin, croissant, cookies, crackers					
Granola					
Bread (white – whole wheat)					
Bagels, tortillas, English muffin					
Pasta, Rice					
Meats					
Livers (chicken, turkey, veal, lamb, beef, pork)					
Beans					
Lentil, Humus					
Milk					
Orange juice					
Potato					
Oat, flour, cornmeal					
Asparagus, Artichoke					
Broccoli, beets, turnip, Brussel sprouts					
Spinach, lettuce, avocado					
Nutritional yeast					
Energy bars, protein bars, sank bars, breakfast bars					Brand?
Complete nutrition drinks (special K, Ensure, Boost, Orgain, Carnation, …. others)					Brand?
Multivitamin supplements, Prenatal supplements					Brand?
Folic acid supplements, B-vitamins supplements					Brand?

Supplemental Table S2: General participants characteristics.

Characteristic	N	Mean (SD)		Reference range:
Age (years)	33	30.80 (4.89)		
Males (%)	21	63.6 %		
BMI (kg/m^2)	33	26.14 (5.35)		Normal weight: 18.5-25
	M (21)	27.9 (5.5)		Overweight: 25-30
	F (12)	23.7 (4.2)	$p = 0.03$	Obese: >30
WHR	33	0.85 (0.08)		Low health risk:
	M (21)	0.88 (0.07)		M < 0.9
	F (12)	0.78 (0.06)	$p < 0.001$	F < 0.85

N, number of participants; **SD**, standard deviation; **M**, males; **F**, Females. **BMI**: body mass index, **WHR**: waist to hip ratio. p-value refer to comparison between M &F.

Supplemental Table S3: Mean systemic markers.

Systemic Markers	Mean (SD)	Normal Rang
Serum folate (µg/L) [a]	14.8 (5.12)	2 - 20
RBC folate (µg/L) [a]	608.32 (168.4)	140 - 628
Homocysteine (µmol/L)	21.41 (7.82)	4 -15
Serum B12 (pg/ml)	459.5 (186.5)	200 - 950
MMA (nmol/L)	125.7 (49.1)	87 - 318
Plasma B6 (µg/L)	18.9 (15.4)	5 - 50
Plasma B2 (µg/L)	20.21 (23.6)	4 - 24
HGB (g/dL)	14.92 (1.81)	**M**: 13.5 -17.5 **F:** 12 - 15.5
HCT (%)	48.1 (5.7)	**M**: 42 - 54 **F:** 38 - 46
RBC (10^6/L)	5.10 (0.6)	**M**: 4.7 - 6.1 **F:** 4.2 - 5.4
WBC (10^9/L)	6.26 (1.68)	4.5 - 11
Neut (%)	51.6 (10.7)	45 - 75
Lymph (%)	36.4 (8.9)	20 - 40
Mono (%)	8.1 (2.3)	2 – 8
MCV (fL)	94.6 (8.7)	80 - 96
MCH (pg)	29.3 (2.1)	23 - 31
MCHC (g/dL)	31.1 (1.74)	32 - 36
RDW-SD (fL)	46.6 (5.6)	39 - 46
PLT (10^3/µl)	264.3 (63.93)	150 - 400
MPV (fL)	11.19 (0.83)	7 – 11

Supplemental Table S4: Nutrient intake.

Nutrient Intake	*Mean (SD)*	*RDA or AI* [c]	
Protein (g)	89.3 (37.2)	0.8 g/kg/day	
Choline (mg)	306.5 (130.5)	M: 550 mg/ day	F: 425 mg/day
Iron (mg)	14.9 (6.8)	M: 8 mg/ day	F: 18 mg/day
Vitamin B1 (mg)	1.31 (0.54)	M: 1.2 mg/ day	F: 1.1 mg/day
Vitamin B2 (mg)	1.22 (0.81)	M: 1.3 mg/ day	F: 1.1 mg/day
Vitamin B3 (mg)[a]	21.2 (10.2)	M: 16 mg/ day	F: 14 mg/day
Vitamin B6 (mg)	1.54 (0.92)	M: 1.3 mg/ day	F: 1.3 mg/day
Vitamin B12 (mcg)	4.94 (12.2)	M: 2.4 mcg/ day	F: 2.4 mcg/day
Folate (mcg)	401.76 (230.9)	M: 400 mcg/ day	F: 400 mcg/day
Folate (mcg DFE) [b]	495.9 (303.8)		

[a] recommendation is expressed as niacin equivalent (NE); [b] recommendation is expressed as dietary folate equivalent (DFE); [C] recommendations are expressed as Recommended Dietary allowance (RDA) or Adequate Intake (AI) for group ages (19-50 yr). n=33.

Supplemental Table S5: Proposed comparison structures.

Structure 1, serum folate tertiles

Tertiles (n)	T1(11)	T2(11)	T3(11)	p- value
Serum Folate (ng/ml)	9.3 (1.9)	14.4 (1.3)	20.6 (2.8)	< 0.0001
RBC Folate (ng/ml)	563.5 (144.7)	571.6 (119.5)	689.9 (210.9)	0.14
Homocysteine (µmol/L)	27.4 (7.2)	20.0 (6.2)	16.8 (6.3)	0.002
Total folate Intake (µg DFE)	576.0 (339.4)	626.3 (336.4)	484.8 (209.4)	ns
Folic acid intake (µg DFE)	204.2 (204.8)	233.7 (169.1)	187.1 (89.6)	<0.0001
FAR	0.31 (0.01)	0.36 (0.01)	0.42 (0.02)	ns

Structure 2, total folate intake tertiles

Tertiles (n)	T1(11)	T2(11)	T3(11)	p- value
Total folate intake (µg DFE)	289.8 (73.4)	490.5 (59.4)	906.9 (237.9)	< 0.0001
Folic acid intake (µg DFE)	98.6 (53.6)	152.2 (39.1)	374.2 (168.3)	< 0.0001
FAR	0.36 (0.03)	0.32 (0.01)	0.41 (0.01)	ns
Serum Folate (ng/ml)	13.9 (4.4)	15.4 (4.8)	15.0 (6.3)	ns
RBC Folate (ng/ml)	637.2 (215.7)	571.3 (145.1)	616.5 (143.6)	ns
Homocysteine (µmol/L)	25.5 (7.7)	19.0 (4.8)	19.6 (9.2)	0.09

Structure 3, folic acid intake tertiles

Tertiles (n)	T1(11)	T2(11)	T3(11)	p- value
Folic acid (µg DFE)	85.6 (35.6)	155.9 (18.5)	383.4 (157.8)	<0.0001
Folate (µg DFE)	362.4 (124.2)	503.9 (240.9)	820.7 (298.1)	<0.0001
FAR	0.24 (0.01)	0.38 (0.02)	0.46 (0.01)	0.003
Serum Folate (ng/ml)	13.9 (4.5)	14.5 (5.2)	15.9 (5.1)	ns
RBC Folate (ng/ml)	583.7 (210)	605.6 (146.2)	635.7 (153.9)	ns
Homocysteine (µmol/L)	24.1 (7.1)	21.4 (8.3)	18.8 (7.8)	ns

Structure 4, folic acid:total folate intake ratio tertiles (FAR).

Tertiles (n)	T1(11)	T2(11)	T3(11)	p- value
FAR	0.2 (0.06)	0.35 (0.04)	0.54 (0.02)	< 0.0001
Folic acid (µg DFE)	99.1 (51)	180.7 (92.5)	345.1 (184.5)	< 0.0001
Folate (µg DFE)	492.0 (253.9)	510.2 (240.4)	684.9 (369.1)	ns
Serum Folate (ng/ml)	13.6 (4.8)	14.5 (5.2)	16.2 (5.5)	ns
RBC Folate (ng/ml)	581.7 (205.8)	625.9 (144.2)	617.3 (168.4)	ns
Homocysteine (µmol/L)	23.2 (7.0)	20.5 (7.8)	20.4 (8.9)	ns

FAR; folic acid; total folate intake ration, DFE; (dietary folate equivalent), Data presented as mean (± SD), n=33. P values denotes significant difference between tertiles.

Supplementary Table S6: Qiagen- RT^2 qPCR Primers

Gene Symbol	Gene name	NM_Number	Band Size	Reference position	Catalog number
MLH1	mutL homolog 1	NM_000249	102	1822	PPH00196F-200
ACTB	Beta Actin	NM_001101	174	730	PPH00073G-200
UNG	Uracil-DNA glycosylase	NM_003362	84	2068	PPH01727E-200
MGMT	O-6-methylguanine-DNA methyltransferase	NM_002412	87	160	PPH01519F-200
MTHFR	Methylenetetrahydrofolate reductase (NAD(P)H)	NM_005957	103	2107	PPH00027F-200
MTR	5-methyltetrahydrofolate-homocysteine methyltransferase	NM_000254	128	3883	PPH10237A-200

HPRT1	Hypoxanthine phosphoribosyl-transferase 1	NM_000194	57	332	PPH01018C-200
